# Breast cancer outcomes in South Asian population of West Yorkshire

**DOI:** 10.1038/sj.bjc.6601795

**Published:** 2004-04-20

**Authors:** G Velikova, L Booth, C Johnston, D Forman, P Selby

**Affiliations:** 1 Cancer Research UK Clinical Centre-Leeds, Cancer Medicine Research Unit, St James's University Hospital, Beckett Street, Leeds LS9 7TF, UK; 2 Northern and Yorkshire Cancer Registry and Information Service, Arthington House, Hospital Lane, Leeds LS16 6QB, UK; 3 School of Medicine, University of Leeds and Northern and Yorkshire Cancer Registry and Information Service, Arthington House, Hospital Lane, Leeds LS16 6QB, UK

**Keywords:** breast cancer, survival, ethnicity, South Asian, treatment delays

## Abstract

Objective: To examine tumour stage at diagnosis, treatment, patient and provider delays to diagnosis/treatment and survival of South Asian patients with breast cancer in Yorkshire in comparison with the general population. Design: Retrospective study, using Yorkshire Cancer Registry population-based data on breast cancer. Data on 16 879 women with breast cancer diagnosed between 1986 and 1994 was available, of which 120 patients were South Asian. All-cause survival, controlling for age, socio-economic profile, tumour stage and treatment was examined. Effects of ethnicity on tumour stage at diagnosis, treatment, patient and provider delays to diagnosis and treatment were described. Over the period 1986–1994, an increase in the number of registered South Asian patients with breast cancer was observed. South Asian patients were significantly younger at the time of diagnosis and presented with larger primary tumours. They received similar treatment to non-Asian patients, but a higher mastectomy rate was noted. South Asian patients' survival, after controlling for age differences was similar to non-South Asian patients. South Asian patients had a significantly longer patient-related delay between initial symptoms and presentation to GP and a slightly longer provider-related delay in time to diagnosis and treatment. In conclusion, outcomes of breast cancer treatment in South Asian patients were similar to non-Asian patients. Asian patients presented later to their GPs, with larger primary tumours and more frequently had mastectomy.

South Asian minority ethnic groups make up to 3% of the population of England (Office for National Statistics, 1996). Cancer is a common cause of death in this population (Bahl, 1996; Wild and McKeigue, 1997). A cross-sectional analysis of mortality by country of birth revealed that although ischaemic heart disease was the leading cause of death in the South Asian population (causing 4230 deaths during the period 1989–1992), lung and breast cancers caused a significant number of deaths (*n*=697) (Wild and McKeigue, 1997).

Research in this area is difficult because data on ethnicity were not routinely collected on hospital records until 1995 and so identification of South Asian patients is problematic. Most often epidemiological studies use country of birth to identify ethnic minority population, recognising that this approach excludes second-generation immigrants. Standardised mortality ratios based on country of birth suggest lower mortality from breast cancer in South Asian women (Wild and McKeigue, 1997; Swerdlow *et al*, 1995). In a study using names to identify South Asian patients in cancer registries, breast cancer incidence in South Asian women was found to be lower than the general population (Winter *et al*, 1999).

Breast cancer remains the most commonly diagnosed female cancer in the UK, accounting for 34% of all cancers in Asian women and 28% in non-Asian (Winter *et al*, 1999). Concerns exist that minority ethnic groups may be getting suboptimal cancer care. For example, the uptake of breast cancer screening is relatively low (Bahl, 1996; Hoare, 1996). Large studies from the USA show ethnic differences in survival after breast cancer, with survival among African-American women significantly worse than white women (Eley *et al*, 1994). The outcomes of breast cancer treatment in ethnic minority groups in the UK have rarely been formally studied (Selby, 1996; McKinney *et al*., 1999). One recent study examined 10 year survival from breast cancer in South Asian women from South East England and found higher relative survival in comparison with the non-South Asian population (Dos Santos Silva *et al*, 2003).

The purpose of this project was to detect possible delays in diagnosis, differences in treatment and in outcomes in South Asian breast cancer patients from Yorkshire, using the Yorkshire Cancer Registry dataset (now part of the Northern and Yorkshire Cancer Registry and Information Services). The hypotheses were based on the extensive studies of outcomes by ethnicity in the USA, as at the time of initiation of the project no data from UK was available. It was hypothesised that the South Asian community in the UK might have poorer survival from breast cancer in comparison with the non-Asian population. The specific aims were to describe the effects of ethnicity on tumour stage at diagnosis, delivered treatment and patient and provider delays to diagnosis and treatment (as a surrogate measure of patient access to hospital treatment). The effects of ethnic group on patient survival were examined, controlling for age, socio-economic profile, tumour stage and treatment.

## PATIENTS AND METHODS

### Identification of Asian patients

We used data from the former Yorkshire Cancer Registry, serving a population of 3. 6 million people for the time period 1986–1994.

South Asian was defined as a person with ethnic origin in either Pakistan, India or Bangladesh. This definition includes Asians from the Indian sub-continent and from East Africa and Sri Lanka whose origins were in the Indian subcontinent. The Registry did not routinely record the ethnic origin of patients and this information was available in only approximately 4% of the cases. Data on religion and birthplace was recorded for 69 and 58% of cases.

Selection of South Asian patients was achieved using a combined strategy of identifying South Asian names, independent selection of cases based on data on ethnic origin, birthplace and religion and final visual inspection of the combined data sets. Initially, the Registry's entire data set was scanned against Nam Pehchan software to identify partial or complete matches against the programme's built-in dictionary of South Asian names (Nam Pehchan computer software Version 1.1, Bradford Health Authority) (Cummins *et al*, 1999). Second, all cases with available and matching ethnic origin (Pakistani, Indian, Bangladeshi), birthplace (Pakistan, Indian, Bangladesh, Sri Lanka, East Africa) and religion (Sikh, Hindu, Moslem, other) were selected from the full list of cancer registrations. The two data sets were matched and the resulting list of names, plus information on birthplace, religion, ethnicity was submitted to two reviewers (GV, LB) for final classification. The reviewers rated the cases independently, using books with lists of names as references (Schimmel, 1989; Patel, 1992). The inter-rater agreement was good with Kappa coefficient 0.84. Cases of disagreement and undetermined cases were reviewed by four colleagues of Asian ethnic origin to achieve consensus.

Once South Asian patients were identified, the Standardised Incidence Rate Ratio comparing South Asian with non-South Asian patients was calculated using population data for Yorkshire from 1991 Census. The result was compared with data from other studies in the UK.

### Demographic and tumour data

Patient age was categorised into four age groups: ⩽49, 50–64, 65–74 and ⩾75 years. In addition, for survival analysis age was split into two groups below and above 50 years. These age groups are frequently used as a surrogate measure of menopausal status. Socio-economic status was characterised using the ward level Carstairs Index scores (Dale and Marsh, 1993). The ward level Carstairs Index scores ranged from −5.13 to 17.63, with a mean score of 0. Positive scores indicate greater levels of deprivation. For analysis purposes, the scores were grouped into the quartiles <−2, −2 to <0, 0 to <3, above 3.

The Registry collects information on tumour size, regional lymph nodes, histologic grade, treatment and patient and provider delays (Sainsbury *et al*, 1999). Tumour size was grouped as ⩽2 cm and >2 cm. Grade was defined as well, moderately or poorly differentiated. Treatment modalities were grouped as surgery (with separate measurement of mastectomy rate and surgeon workload), radiotherapy, hormonotherapy and chemotherapy. The Yorkshire Cancer Registry records key dates of first symptoms, GP referral, first visit at hospital and start of definitive treatment. We calculated the time from the symptom date to GP referral to reflect patient-related delay (groups ⩽60 and >60 days) and the time from GP referral to the first hospital visit (⩽14 and >14 days) and from the first hospital visit to the definitive treatment (⩽14 and >14 days) to reflect provider delay. The cut-off points were selected to reflect the pattern of data, current standard of cancer care and systematic reviews of effects of delays on breast cancer outcomes (Sainsbury *et al*, 1995).

As the years of the study included 1988 when the NHS Breast Screening Programme was introduced, information was collected on number of patients screened.

Survival was calculated from the date of registration. Patients still alive at the time of the study were censored at 1st January 1999. Initial inspection of survival data revealed a higher proportion of censored data in the Asian patients in comparison with non-Asian for several cancer sites, including breast (69% censored data *vs* 52%, respectively). Additional active follow-up was performed for all Asian patients and for a random sample of non-Asian patients. Patient status was checked at the corresponding Health Authorities, and if still ‘alive’ letters were sent to GPs with a request for information. The local Ethics committee gave permission to contact patients' GPs. After the active follow-up, definitive information on patient status was obtained for 94% of Asian breast cancer patients and for 98% of non-Asian patients.

### Statistical analysis

Analyses of association between ethnic groups and demographic, tumour and treatment variables were performed using frequency tables and *χ*^2^ statistic. When calculating *χ*^2^ statistic cases with missing values were excluded. Mean age of Asian and non-Asian patients was compared with a *t*-test.

Previous research suggests that variations in the quality of cancer care between health districts account for late diagnosis, treatment and outcome differences (Sainsbury *et al*, 1995). Socio-economic differences in breast cancer survival have been reported for England and Wales (Coleman *et al*, 1999). Therefore, logistic regression was used to control for the possible effects of socio-economic status, hospital district and patient age, when analysing the effect of ethnicity on cancer stage at diagnosis and treatment modalities. The models included cancer stage (or treatment) as the dependent binary variable and socio-economic status, hospital district, patient age and ethnicity (South Asian *vs* non-Asian) as independent variables.

Survival curves were estimated by the Kaplan–Meier method and compared using log-rank test. Cox's proportional hazards regression analysis was used to compare survival by ethnicity, with allowance for other factors – age, tumour size/nodal status, tumour grade and treatment.

Significance level was set at 1% to allow for multiple comparisons. The analyses were performed with SPSS Version 11.0 for Windows (SPSS Inc, Chicago, IL, USA) and SAS version 6.07 (SAS Institute Inc, Cary, NC, USA).

## RESULTS

### Breast cancer registrations, socio-demographic and tumour characteristics

Information on 16 879 patients with breast cancer from 1986 to 1994 was available and 120 (0. 7%) were identified as South Asian. The Standardised Incidence Rate Ratio in this study comparing South Asian with non-South Asian patients was 0.56 (95% CI 0.46–0.66).

From the ethnic minority patients 31% lived in Bradford area, 24% in Leeds, 18% in Dewsbury and 12% in Huddersfield.

Descriptions of the demographic, socio-economic and tumour characteristics of the patients, stratified by ethnicity are presented in Table 1

**Table 1:** Demographic, socio-economic and tumour characteristics of the patients stratified by ethnicity

	**Non-Asian**		**Asian**			
**Variables**	**Number**	**Percent**	**Number**	**Percent**	***P*-value^a^ (*χ*^2^ statistic)**	***P*-value^b^ logistic regression)**
**Year period**						
1986–1988	5069	30	24	20	0.004	—
1989–1991	5756	34	39	33		
1992–1994	5934	35	57	48		
Total	16 759	100	120	100		
						
**Age (years)**						
<50	3683	22	66	55	<0.0001	—
50–4	5731	34	33	28		
65–74	3661	22	16	13		
75+	3684	22	5	4		
						
**Carstairs Index**						
< −2 (affluent)	4177	25	9	8	<0.0001	—
−2 to <0	4252	26	14	12		
0 to <3	3810	23	16	13		
3+(deprived)	4254	26	81	68		
Unknown	(19)	(0.1)				
**Tumour size**						
⩽2 cm	3187	56^c^	19	35	0.002	0.002
>2 cm	2528	44	35	65		
Unknown	(11044)	(66)^d^	(66)	(55)		
						
**Involvement of regional lymph nodes**						
Negative nodal involvement	2591	34^c^	14	22	0.054	0.45
Known involvement	5083	66	49	78		
Unknown	(9085)	(54)^d^	(57)	(48)		
						
**Tumour grade**						
Well	1190	13^c^	4	5	0.028	0.08
Moderately	2810	31	23	27		
Poorly differentiated	5015	56	57	68		
Unknown	(7744)	(46)^d^	(36)	(30)		

a *P*-values from *χ*^2^ statistic, excluding cases with missing data.

b Logistic regression controlling for age, hospital district and Carstairs Index.

c Percentage calculated on the basis of available data only.

d Proportion of missing data.

. In 1986–1994, there was an increase in the number of registered South Asian patients with breast cancer. The rate of increase is higher than for non-Asian women (*P*<0.004). Asian women were significantly younger than non-Asian at the time of diagnosis of breast cancer (mean age 49.7 *vs* 62.0 years, respectively, *P*<0.0001), with a greater proportion being less than 50 years old.

A significantly larger proportion of South Asian breast cancer patients belonged to the deprived socio-economic groups (Carstairs Index above 3). A significantly higher proportions of South Asian patients presented with tumours larger than 2 cm. The differences remained after controlling for socio-economic status, age and hospital district (Table 1). The proportions of patients with involved regional lymph nodes and with poorly differentiated tumours were not significantly different. The differences in tumour-related variables should be interpreted with caution due to the large amount of missing data.

The Registry data showed that 7.5% (*n*=9) South Asian patients 8.8% (*n*=1469) non-Asian patients, diagnosed with breast cancer, participated in breast screening. The information on breast screening was not included in any further analysis due to the small number of South Asian patients.

## Time to GP presentation, referral pattern and treatment (Table 2)

**Table 2:** Time to GP presentation, referral pattern and treatment

	**Non-Asian**		**Asian**			
	**Number**	**Percent**	**Number**	**Percent**	***P*-Value^a^ (*χ*^2^ statistic)**	***P*-value^b^ (logistic regression)**
**1st symptom to gp presentation**						
⩽60 days	3734	66^c^	16	47	0.017	0.005
>60 days	1889	34	18	53		
median (days)	31		61			
Unknown	(11136)	(67)^d^	(86)	(72)		
						
**GP referral to 1st hospital visit**						
⩽14 days	7127	64^c^	49	52	0.049	0.028
>14 days	4062	36	46	48		
median (days)	11		14			
Unknown	(5570)	(33)	(25)	(21)		
						
**1st hospital visit to treatment**						
⩽14 days	10322	63^c^	47	39	<0.0001	<0.0001
>14 days	6113	37	72	61		
median (days)	9		14			
Unknown	(144)	(0.9)	1	(0)		
						
**Treatment**						
						
**Any surgery**						
Yes	14034	84	111	93	0.08	0.16
No	2711	16	9	8		
Unknown	(14)	(0)	(0)	(0)		
						
**Mastectomy rate**						
Yes	8155	49	75	63	0.003	0.007
No	8604	51	45	38		
Unknown	(0)	(0)	(0)	(0)		
						
**Radiotherapy**						
Yes	8119	49	63	53	0.57	0.37
No	8444	50	57	48		
Unknown	(194)	(1)	(0)	(0)		
						
**Chemotherapy**						
Yes	2566	15	38	32	<0.0001	0.15
No	13934	83	82	68		
Unknown	(258)	(2)	(0)	(0)		
						
**Hormonotherapy**						
Yes	12862	77	93	78	0.59	0.01
No	3752	22	27	23		
Unknown	(145)	(1)	(0)	(0)		

a *P*-values from *χ*^2^ statistic, excluding cases with missing data.

b Logistic regression controlling for socio-economic status, age and hospital district.

c Percentage calculated on the basis of available data only.

d Proportion of missing data.

All patients had their first hospital visit date recorded, 67% had GP referral date (*n*=11 285) and 44% of the patients (*n*=7433) had symptom date recorded. Asian patients had longer period between the onset of symptoms and GP presentation (median of 61 days *vs* a median of 31 days for non-Asian women, *P*=0.005 after controlling for age and hospital district) (Table 2). Controlling for socio-economic status did not explain the ethnic differences in presentation (data not shown). There was no significant difference in the period between the GP referral and the first hospital visit, using the *a priori* stated definition of *P*<0.01. Significantly larger proportion of non-Asian patients had definitive treatment within 2 weeks of first hospital visit (*P*<0.0001). However, the median time to treatment in South Asian patients was only 14 *vs* 9 days in non-Asian patients. A difference in the order of less than 1 week is unlikely to have an impact on treatment results (Table 2).

The use of any surgery was similar in Asian and non-Asian patients, but a significantly higher mastectomy rate was found for Asian patients, after controlling for age and district of residence (to account for regional differences in surgical practices). As tumour characteristics like size and nodal status are expected to influence treatment, an additional subgroup analysis was performed comparing the mastectomy rate in node-positive patients and in patients with tumours larger than 2 cm, controlling for age. Mastectomy rate did not differ significantly between South Asian and non-Asian patients who had large tumours or positive lymph nodes. Therefore, ethnicity in itself did not appear to be associated with different surgical treatment. These results should be interpreted with extreme caution due to the large amount of missing data on nodal status and tumour size. Only 49 South Asian patients had positive lymph nodes and only 35 South Asian patients had tumours larger than 2 cm. However, it appears that the higher mastectomy rate most likely reflects more advanced tumours at presentation, requiring radical surgery, rather than different treatment approach to South Asian patients.

The use of radiotherapy was similar in both groups. A larger proportion of Asian patients received chemotherapy, but after adjustment for age and district the differences were not significant. The use of hormonotherapy treatment appeared similar in both groups, but after adjustment for age and hospital district a significantly higher use of hormonotherapy was noted in the Asian patients.

### Survival

**Figure 1:**
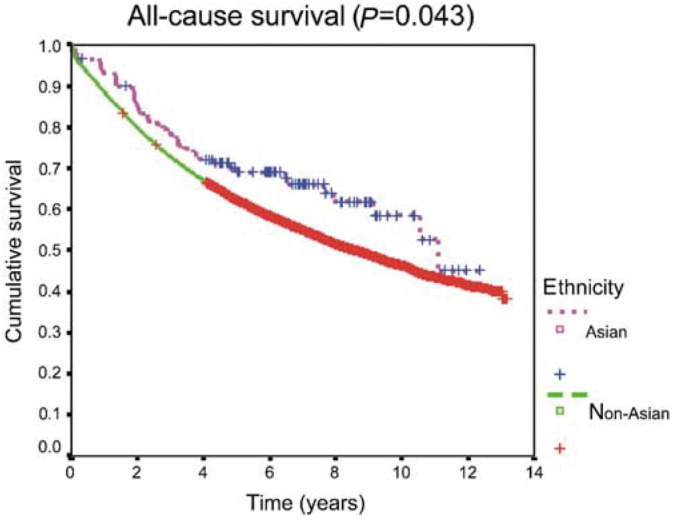
All-cause survival curves by ethnicity

In the univariate analysis, a marginally favourable survival advantage was observed for the Asian patients in comparison with non-Asian (Figure 1

, p=0.043). However, examination of survival by age groups (<50 *vs* ⩾50 years) revealed no significant differences. The small differences observed for all patients seemed related to the larger proportion of young patients in the ethnic minority group. The multivariate analysis (Table 3

**Table 3:** A 5-year relative risk of death among South Asian breast cancer patients compared with the general population, unadjusted and after adjustment for demographic, tumour and treatment-related variables (Cox Proportional Hazards model)

**Adjustment**	**Relative risk of death for South Asians**	**95% CI**	
None (ethnicity alone)	0.74	0.54	0.99
Plus age	1.01	0.75	1.36
Plus tumour size, grade	0.75	0.55	1.01
Plus treatment	0.87	0.64	1.17
Plus workload	0.78	0.58	1.05
Plus carstairs index	0.68	0.50	0.91
			
Plus age	1.01	0.75	1.36
Plus age, tumour size, grade	0.98	0.71	1.33
Plus age, tumour size, treatment	0.97	0.71	1.29
Plus age, tumour size, treatment, consultant workload	0.95	0.71	1.29
Plus age, tumour size, treatment, consultant workload, carstairs index	0.89	0.66	1.21

) confirmed that patient age explained the small difference in overall survival in favour of Asian patients. Controlling for socio-economic deprivation suggested that South Asian patients living in deprived areas had better survival, compared to non-Asian patients. However, when age was included in the model, controlling for the other case mix factors (disease and treatment characteristics) did not have an effect on survival. In a model including all possible explanatory variables, only controlling for age changed the estimates, thus suggesting that the small difference in survival of South Asian patients can be explained by their younger age and not by any of the other variables (socio-economic deprivation, tumour stage or treatment) (Figure 2

**Figure 2:**
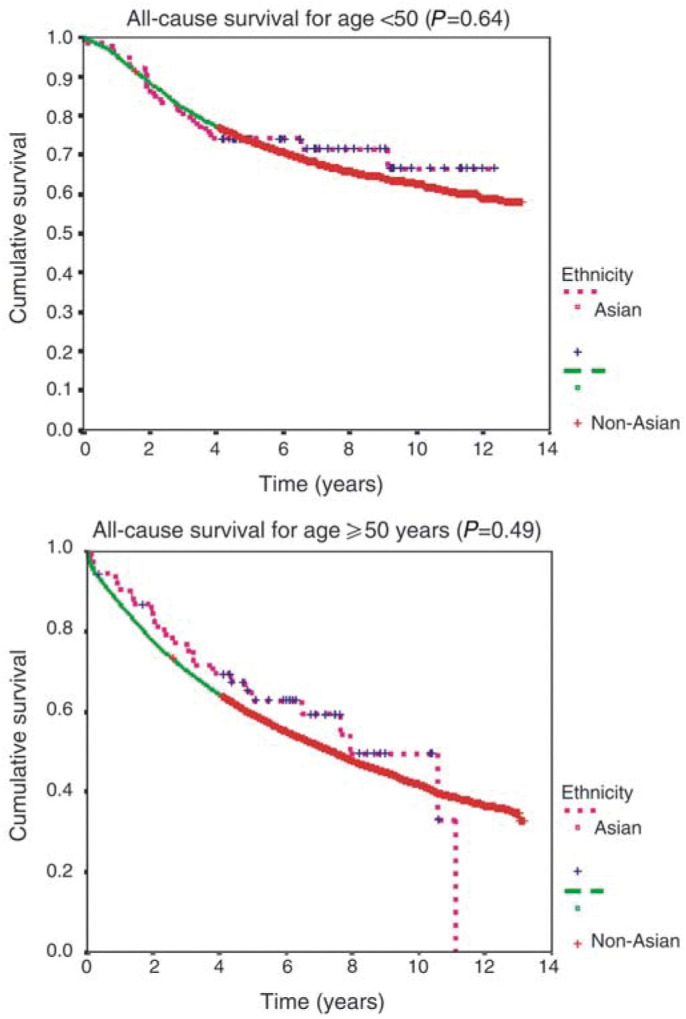
All cause survival curves by ethnicity and age

).

## DISCUSSION

This study described the state of breast cancer in the Yorkshire South Asian population in terms of socio-demographic characteristics of patients, stage of disease, treatment and outcomes, including long-term survival. Over the period 1986–1994, there was an increase in the number of registered South Asian patients with breast cancer. These results are consistent with other studies, showing increased incidence rates of breast cancer in South Asian women over time, while the rates decreased among non-Asian population (Winter *et al*, 1999; Dos Santos Silva *et al*, 2003; Smith *et al*, 2003). These changes could be explained by the ageing population of South Asians, increased proportion of second-generation immigrants and significant differences in lifestyle and other exposures occurring in South Asian minority population in the UK.

South Asian patients were significantly younger at the time of diagnosis. This finding is likely to reflect the generally younger age of ethnic minorities in Britain (Office for National Statistics, 1996). It is consistent with observations in ethnic minority groups both in the UK and the USA (African-American women) (Coates *et al*, 1992; Elmore *et al*, 1998; Joslyn and West, 2000; Dos Santos Silva *et al*, 2003; Smith *et al*, 2003).

South Asian women presented with larger tumours, with the difference remaining statistically significant after adjustment for age and hospital district. Similar observations were reported from research in the USA (Elmore *et al*, 1998; Hunter, 2000). The trend for more advanced stage at diagnosis is consistent with the longer interval found between first symptoms of breast cancer and presentation to the GP. The observed higher mastectomy rate also suggests that more radical surgery may have been required to achieve adequate local control. These findings are consistent with other research showing lower uptake of breast screening programmes by ethnic minority populations (Hoare, 1996). Possible cultural differences and different attitudes to cancer could account for these important observations (Bottorff *et al*, 1998; Lannin *et al*, 1998; Tam *et al*, 2003). Interventions aimed at increasing breast cancer awareness and encouraging breast screening could be recommended.

After controlling for age, survival of South Asian women was essentially similar to non-Asian breast cancer patients. No significant effect was found for any of the case-mix factors studied (including socio-economic status, disease stage at presentation, treatment and consultants work load). Our findings are similar to those of Dos Santos Silva *et al* (2003), who found higher relative survival of South Asian women with breast cancer (RR 0.82, 95% CI 0.72–0.94, *P*=0.004), and adjustment for age at diagnosis reduced the magnitude of the difference (RR 0.88, 95% CI 0.78–1.01, *P*=0.07). However, in their study adjustment for stage and socio-economic deprivation strengthened the survival difference.

In contrast to these findings from UK, extensive research from the USA suggested significantly poorer survival in African Americans (Eley *et al*, 1994). However, in a study comparing breast cancer patients who had equivalent stage disease and were treated uniformly in a single institution, race did not influence outcome (Heimann *et al*, 1997). Furthermore, there is a documented survival advantage of Japanese patients and equivalent survival of Chinese patients compared to Caucasians and these findings remain unexplained (Clegg *et al*, 2002; Tam *et al*, 2003).

No clinically meaningful differences were identified in provider-related delay between the South Asian and the non-Asian patients with breast cancer. Furthermore, the recorded data on definitive treatment for breast cancer showed no significant differences in the active anticancer treatment, apart from a higher mastectomy rate. Therefore, this study did not support the hypothesis that Asian patients may have poorer access to hospital treatment, but a concern remains about the more advanced stage of disease at presentation. These results are difficult to compare with other data on ethnic minorities in the UK and further research should be encouraged.

Several limitations of the present study should be discussed, particularly in relation to the use of retrospective data from Cancer Registry and the methods for selection of South Asian patients. As routine collection of data on patient ethnic origin started only in 1995, we had to use alternative methods for identifying South Asian patients with breast cancer. A rigorous procedure for selection of South Asian patients was employed following suggestions from other researchers (Cummins *et al*, 1999). However, the possibility of error due to the software not identifying rare South Asian names or to the large amount of missing data on birthplace and religion cannot be excluded. Nevertheless, it is our belief that this approach was the only feasible way of identifying South Asian patients in order to conduct this study. As an external validation of this approach, the standardised Incidence Rate Ratio was calculated 0.56 (95% CI 0.46–0.66), which was found to be similar to the rate, reported by Smith *et al* (2003) (0.61, 95% CI 0.50–0.73) and Winter *et al* (1999) (0.64, 95% CI 0.59–0.69). It should be noted that we identified 0.7% of registered breast cancer cases as South Asian, whereas other authors identified slightly higher proportions – Dos Santos *et al* (2003) identified 1.9% of breast cancer registrations as South Asian, Winter *et al* (1999) – 1.3%.

The use of retrospective Registry data had the advantage of a large population-based database, but the disadvantage of not having complete staging information on all cases and no records of prognostic information, such as hormone receptor status. When examining survival, hormone receptor status is an important prognostic factor and the lack of that information may potentially lead to erroneous conclusions. The missing data on tumour size and lymph nodal status makes the interpretation of the results difficult and cautious. Although the Registry database of breast cancer patients was large (above 16 000 cases), the number of South Asian patients identified was relatively small (only 120), which made the investigation of the effect of various case-mix factors difficult.

The study raised some concerns about the validity of death information among South Asian women, derived from Office of National Statistics data. Initial observations showed significantly higher proportion of censored cases for South Asian patients with all types of cancer. This led to the hypothesis that terminally ill patients may return to their country of birth and their deaths would not be recorded. This hypothesis was explored by active follow-up of all South Asian patients, and for comparative purposes a random sample of non-South Asian patients. For the breast cancer cases, the active follow-up did not contribute significantly more information and following this rigorous quality assurance procedure, the survival data for the breast cancer patients can be considered reliable.

In conclusion, South Asian patients with breast cancer were found to have more advanced disease at diagnosis and possibly to present later to their GP when they had breast symptoms. The multimodality treatment of breast cancer and the outcomes of treatment and survival were similar to those of non-Asian patients. The limitations of using a retrospective data set from Cancer Registry should be recognised. However, large prospective national studies would be difficult and expensive to perform and alternative strategies should be considered. Repeated retrospective analyses at regular intervals (i.e. every 10 years), using existing Cancer Registries databases can be performed to detect changes in the pattern of presentation, treatment and outcomes of cancer in ethnic minorities.
